# Preferences for Tailored Support – Patients’ and Health Care Professionals’ Experiences Regarding Symptoms and Self-Management Strategies During the First Year After Curatively Intended Prostate Cancer Treatment

**DOI:** 10.2147/PPA.S440689

**Published:** 2024-02-02

**Authors:** Nazmije Kelmendi, Marie Nilsson, Marina Taloyan, Kay Sundberg, Ann Langius-Eklöf, Åsa G Craftman

**Affiliations:** 1 Department of Neurobiology, Care Sciences and Society, Division of Nursing, Karolinska Institutet, Stockholm, Sweden; 2 Academic Primary Health Care Center, Region Stockholm, Stockholm, Sweden; 3 Department of Neurobiology, Care Sciences and Society, Division of Family Medicine and Primary Care, Karolinska Institutet, Stockholm, Sweden

**Keywords:** prostate cancer, self-management, symptoms, support, survivorship, information

## Abstract

**Purpose:**

There is an increase in the number of men undergoing screening for prostate cancer, and advancements in treatments, which implies current knowledge about symptoms and self-management. This study aims to explore experiences of symptom distress, and self-management strategies during the first year after curatively intended treatment for prostate cancer, as identified by patients and health care professionals.

**Methods:**

A qualitative design was used, including data triangulation from individual interviews with patients (n =17) and one focus group interview with healthcare professionals (n =5). Thematic analysis was used.

**Results:**

The two main themes were identified: *living with the consequences of treatment* and *navigating a new situation. Living with the consequences of treatment* illustrated how losing control of bodily functions such as bladder, bowel, and sexual functions interfered with daily life. A stigma around the disease was described, and a life living in an unfamiliar body challenged ideas of masculinity. The first months after treatment ended was a distressing period related to the abruption in frequent contact with healthcare providers, and concerns about the future. The second theme, *navigating a new situation*, illustrates that self-management strategies varied, due to individual factors as did the need for tailored information and support provided from healthcare professionals and family, which was highly valued. Information and support were described as complex topics and healthcare professionals emphasized the need for appropriate education for staff to provide proper support to men after ended treatment.

**Conclusion:**

Lingering symptoms and concerns were evident during the first year after treatment. Self-management strategies varied, and timely and tailored information and support during the first year were considered highly valued, important, and preferred by patients. Our results indicate that support should be offered immediately after curatively intended treatment.

## Introduction

Prostate cancer (PC) is the most common cancer in men in Europe and the second-most common cancer worldwide.^1^ There is increasing prevalence due to an ageing population, diagnoses made at an earlier stage, and advances in treatment.^2^ There are different treatment options for different cancer stages, such as watchful waiting and active surveillance, and the curatively intended treatments involve surgery or radiation therapy (external beam radiation therapy and brachytherapy), sometimes combined with hormone therapy.^3^ In Sweden, the number of men undergoing opportunistic screening is increasing, and the male population between 50 and 70 years have been offered opportunistic screening, depending on region.^4^ Recently, the European Union recommended that all countries thoroughly evaluate organised screening as there may be a risk of overdiagnosis and overtreatment.^5^

It is well-known that PC treatment can cause long-term concerns and symptoms that may negatively impact a patient’s life after ending treatment.^6^,^7^ Furthermore, patients report long-term unmet informational, physical, and emotional needs.^8–10^ Undiagnosed symptoms impact the quality of life and recovery, which impose early identification for timely support.^11^ Symptom management is an important aspect of cancer which can improve overall well-being, health and quality of life.^12^

After ending curatively intended treatment, patients have outpatient follow-ups, including at least two prostate-specific antigens (PSA) measurements during the first year.^3^ In Sweden, physician appointments are generally scheduled three months after treatment ends, and patients are encouraged to contact their cancer nurse when needed.^13^ Hence, most of the time during this phase, patients are at home and left to manage concerns and symptom distress by themselves,^14^ which implies finding and utilising specific and relevant information.^15^

The most prevalent symptoms in the first year after curatively intended treatment are urinary, bowel, and sexual dysfunction.^16–20^ Other symptoms include fatigue,^19^,^21^ mental health issues such as anxiety and depression^22^,^23^ and hot flashes among men who have undergone hormone therapy.^24^ Strategies for self-management are described as appreciating life, returning to activities or work,^25^ accepting the situation, and dealing with side effects.^26^ Most studies are on a group level and do not combine experiences of symptoms and self-management strategies. It has been suggested to include experiences from patients as well as healthcare professionals in order to gain a comprehensive understanding of patients’ needs.^27^ Considering the increase in screening and advances in treatments for PC, it is essential to gain topical knowledge of how patients and healthcare professionals view of the consequences from treatment.

Therefore, this study aims to explore experiences of symptom distress, and self-management strategies during the first year after curatively intended treatment for PC, as identified by patients and healthcare professionals.

## Methods

### Design

This qualitative study used data triangulation from individual patient interviews and a focus group interview with healthcare professionals (HCP).^28^ The consolidated criteria for reporting qualitative research (COREQ) was used.^29^

### Individual Patient Interviews

#### Setting and Sample

Inclusion criteria were patients diagnosed with PC who were in their first year after the end of curatively intended treatment, spoke Swedish, had no cognitive impairment, and lived in the Stockholm region. Patients were recruited at a specialized clinic at Karolinska University Hospital in Stockholm, Sweden, serving the region’s rural and urban areas. Patients were recruited in two ways: 1) by two nurses working at a urology and oncology clinic and 2) from one post-PC surgery group meeting at the hospital. With the intention of a variation among the participants, the sample was purposefully selected regarding the type of treatment and time after the end of treatment. The two nurses sent invitation letters sequentially to patients with written information about the study, registration of interest to participate, and a pre-addressed and stamped reply envelope. Of 25 invited patients, 14 chose to participate. No reminders were sent. At the post-surgery group meeting, the first and the last author gave seven patients verbal and written information about the study. Three patients contacted the first author afterwards and chose to participate. In total, 17 patients of 32 invited agreed to participate, and there were no dropouts after inclusion.

#### Data Collection

Individual semi-structured patient interviews were performed between October 2019 and June 2020 and lasted, on average, 47 minutes (range: 29–78 minutes). The first author conducted the interviews at a time and place according to the patient’s wishes; no compensation was provided. Eleven face-to-face interviews were performed (four at patients’ homes, two at patients’ workplaces, and five at the interviewer’s worksite). Six interviews were performed by telephone due to COVID-19 pandemic restrictions. The interviewer was not known to or had any relation to the participants. The interview guide was developed within the research group and focused on patients’ experiences the first year after curative intended treatment. The interview guide was tested in a pilot interview with one patient. The guide was found to be expedient; therefore, no changes were made, and the interview was included. Each interview started with the open question, “Can you please tell me how you experienced the time after your treatment and how do you feel now?” followed by questions regarding symptoms, side effects, the support they had received, information needs, and self-care (Table 1).

**Table 1:** Interview Guide - Patients

Questions	Follow-Up Questions
Can you please tell me how you experienced the time after your treatment and how do you feel now?	Problems/symptoms now/since the end of treatment?
Which symptoms/side effects have been difficult to manage after your treatment?	Give examples of self-management strategies. How have you managed with these? Influence on everyday life? Unexpected?
What support have you received from the healthcare system/society?	Emotional, informational, practical? What do you lack/desire? Influence on own finances?
What information have you received from the healthcare system, and from whom?	What do you lack/desire? To whom do you turn to when needed? Do you understand the information? Specific about self-care?
Have you searched for information besides the information you received from healthcare via, for example, the Internet?	How? Useful/meaningful? If not, why not
Is there anything else you want to add that we have not discussed?	

### Focus Group Interview with Healthcare Professionals

#### Setting and Sample

A purposeful sample of healthcare professionals of different professions working at a cancer rehabilitation center in Stockholm were invited to participate in the study. The inclusion criteria was experience working with patients with PC during the first year after treatment end. Initially, the first author approached and informed the chief about the study, who later informed the team. The whole team was invited to participate, however, due to a heavy workload, not all could participate. In total, five HCP participated in the focus group interview: two registered nurses, one psychologist, one occupational therapist, and one dietitian, all specialized in cancer rehabilitation. The HCP worked as a team daily and were previously known to each other. They were, however, unknown to the patients participating in this study.

#### Data Collection

The focus group interview with HCP was performed in February 2020 and was facilitated by a moderator (first author) and an observer (last author) not previously known to the participants. The interview was performed at their workplace in a conference room according to the team’s wishes and lasted 39 minutes. The interview guide was developed by the research group and focused on participants’ professional experience of the patient situation in the first year after completing the curatively intended treatment. The interview started with the question, “What symptoms do patients usually have/can have at different time points after the first year following the end of treatment?” (Table 2).

**Table 2:** Interview Guide- Focus Group

Questions	Follow-Up Questions
What symptoms do patients usually have/can have at different time points after the first year following the end of treatment?	Physical, social, psychological, emotional, existential? When and how often? What help can they get? Referral to other professionals?
What self-care advice do you give to patients?	
How do/can the patients search for information from your experience?	Where can they search for information themselves?
What do you think would be valuable, based on your experience, from a patient perspective, to follow up after the treatment?	Symptoms/concerns/self-care?
Is there anything else you want to add that we have not discussed?	

### Data Analysis

**Figure 1:**
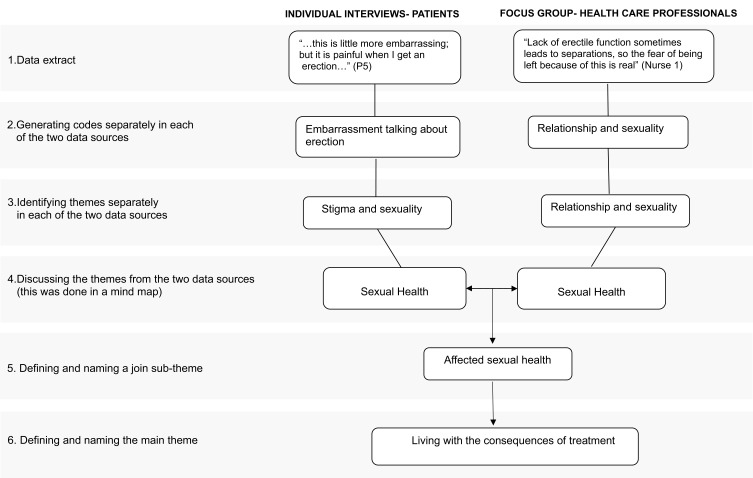
Example of Analysis Process.

The individual and focus group interviews were digitally recorded with the informants’ permission and transcribed verbatim by the first author. Transcribed interviews were read by the first and last author while listening to the recording to check for accuracy. After this, the individual patient interviews and the focus group interview were analyzed separately using thematic analysis, as described by Braun and Clark.^30^ First, the text was read several times to understand its content, and data related to the study’s aims were extracted and transferred to a coding file. Afterwards, codes were generated based on the extracted data, and patterns among codes were identified and interpreted to generate themes. The reviewing process involved relating themes to the text and checking data and codes involving all authors. During the analysis process, the data related to the dataset was checked, and themes were defined and redefined until all authors considered them to be relevant. The separate subthemes generated from the two data sources were analyzed together in a final step. A mind map was created to link the two sources’ identified themes’ similarities, differences, and patterns. New subthemes were extracted, and themes were named and defined (Figure 1). Quotations were chosen from interviews with patients and healthcare professionals to illustrate the findings. Analyses from the interviews with patients and HCPs are presented in two themes and seven subthemes (Table 3). The study participants did not see the transcripts or provide feedback on the findings.

**Table 3:** Subthemes and Themes

Subthemes	Themes
Losing control of bladder and bowel Affected sexual health Living in an unfamiliar body A period of emotional distress	Living with the consequences of treatment
Variations in handling side-effects Formal support and unmet needs Informal source of support	Navigating a new situation

### Ethical Considerations

This study has been performed in accordance with the Declaration of Helsinki.^31^ Ethical approval for the study was obtained from the Swedish Ethical Review Authority (DNR: 2019–00379). Patients and HCP received information about the study, including the possibility that anonymized citations of responses might be published, voluntary participation, and the right to withdraw at any time. Written and oral informed consent was obtained from each participant, and each transcribed interview was coded with a number to ensure confidentiality.

## Results

The median age of the patients was 73 (range 56–80 years). Eight of the patients had undergone surgery, and nine had undergone radiotherapy. Sixteen patients lived with a partner, eight had a university education, and 13 were retired (Table 4). All the health professionals were female, and their work experience with cancer rehabilitation ranged from 2 to 42 years (median 20 years).

**Table 4:** Characteristics of Participating Patients (N =17)

Characteristics of Participating	Patients (N =17)
**Age in years (Range 56–80)**	
Median	73
Inter Quartile Range	67–78
**Partnership**	
Single	1
Married or having a partner	16
**Education level**	
Primary school	1
College	8
University	8
**Employment**	
Retired	13
Employed	4
**Treatments**	
Radical Prostatectomy	8
Radiotherapy /brachytherapy Combined with hormonal treatment	9
**End of treatment in months at the interview (Range 1–15)**	
Median	4
Inter Quartile Range	2–9

The findings are presented in two overarching themes: *Living with the consequences of treatment* and *Navigating a new situation*, with four and three subthemes, respectively.

### Living with the Consequences of Treatment

This theme describes the first year of living with the consequences of treatment, including physical and mental changes.

#### Losing Control of Bladder and Bowel

The most common symptoms of distress described by patients and HCP were loss of control over urinary and bowel functions. Patients described urinary dysfunction symptoms, such as urinary retention, a sudden urge to urinate, leaking urine, or involuntary leaking of urine. Urinating several times during the night affected the patient’s sleep and daily life due to tiredness. Bowel dysfunction was described by patients as a rather unexpected side effect compared to urinary dysfunction and was mostly experienced by patients who had undergone radiotherapy. Bowel dysfunction was exemplified as leakage of faeces, flatus, constipation, or obstructed defecation. Sudden and uncontrolled leakage of faeces could occur during the patients’ daily activities. Both patients and HCPs described how urine and bowel dysfunction negatively impacted spontaneity in everyday life and contributed to withdrawing from activities where it could be challenging to find a toilet.

It is difficult when I wet myself. I avoid going to the cinema because I must bring a change of clothes and stuff like that in a backpack. I do not want to stand in a cinema toilet and change my wet pants. (P8)

#### Affected Sexual Health

Patients and HCPs described the impact of sexual health as an important but sensitive topic to address. HCP explained how the loss of erectile function was the most common and distressing symptom. The patients exemplified negative impact as painful erections, retrograde ejaculation, impotence, and lost desire for sexual intimacy. Despite sexuality being a source of joy and pleasure in life, it was subordinated to survival. Some patients described a deeper relationship with their partner without sexual intercourse.

…it has not come back (sexual function), but you can almost count on that, they cut a little here and there, and across. I do not know if it recovers over time. I will have to talk to the doctor about that later. But it is not the most important thing in this life. (P4)

The patient, without a current partner, expressed thoughts regarding an eventual future relationship and hoped to find a partner understanding of their situation.

The HCP voiced how impotence could radically affect a patient’s life and how they viewed their future. This was especially evident in younger patients, where sexual dysfunction and fertility were exemplified as a cause for divorce.

#### Living in an Unfamiliar Body

The patients experienced bodily changes; for example, weight gain or weight loss, decreased muscle mass, breast enlargement, hot flashes, increased sweating, lymphoedema (eg, swelling in the stomach, ankles, legs, and around the pelvis area), smaller penis after prostate removal, lack of energy, and tiredness. Patients described a lack of energy, particularly during the first months after treatment ended, which was described as not being able to perform activities they had previously done, such as climbing stairs or cutting wood. Overall, the patients voiced how bodily changes had led to perceptions of loss of masculinity and living in an unfamiliar body. The hormone treatments were described as “female hormones” and affected their self-perception as a man. HCP had met patients that described themselves as “mutilated”.

… I think the side effects from the female hormone are disturbing. I have gained 4kg in weight, I have lost hair, my chest and stomach are as bare as a baby’s butt, feels strange, the testicles and penis have become so small, it is difficult to urinate, it is all a bit strange. (P11)

#### A Period of Emotional Distress

Some patients expressed positive thoughts about the future and seized moments in life. For some patients, the symptom distress decreased, but for some patients, concerns regarding future potential side effects and fear of new cancer were present. It was common to observe symptoms like a nodule or pain vigilantly. A younger man, 56 years of age, expressed that it was difficult to have a PC at such a young age. The most distressing period was around three months after the end of treatment and before the first follow-up, while they were waiting for the first post-treatment PSA test result.

The worst thing would be that I have received treatment, but cancer is still there. imagine that…you can only ask how much time I have left… (P9)

The HCP mainly described their experience working with different crisis reactions among prostate cancer patients, like a delayed reaction to the diagnosis and being aware of the risk and fear of death. HCP also described that a crisis could occur when returning to everyday life.

### Navigating a New Situation

This theme describes that after treatment ends, patients need to find information sources that support them in managing and navigating their new life situation. Some sources for information and support were provided by healthcare providers (formal), and some were provided by family and friends (informal).

#### Variations in Managing Side-Effects

Patients described different strategies to manage urine incontinence; for example, sitting and urinating because of the shortened penis or difficulty urinating while standing, urinating even if they did not feel they had to, restricting their fluid intake, and knowing about the nearest toilet before leaving home. Some patients had learned to use incontinence protection depending on activity, eg before drinking a beer, or just as a precaution. In contrast, other patients described insecurity about using incontinence protection correctly and therefore did not find them helpful. HCP described that some patients used paper towels in their underwear. Other patients did not know or had forgotten the right to subsidized incontinence protection and therefore bought it themselves privately at a considerable expense. Another strategy patients described was the regular performance of pelvic floor exercises, but some patients were still unsure whether they performed them correctly while others forgot to do them.

Bowel dysfunctions were handled differently; some contacted healthcare providers for advice and support, changed their eating habits, and tried prescribed medications. Others just accepted the symptoms and did not do anything about them. HCP exemplified poor adherence describing how patients had been prescribed drugs for bowel dysfunction but due to lack of information the drugs were not taken properly or at all.

I have not handled it at all. I do not know who to call about what I have received. I have not taken medicine so I can get a harder stool. I do not know if it helps, I have not picked up that medicine from the pharmacy yet, I am thinking of doing it. (P10)

Patients described handling sexual dysfunction differently: trying to masturbate, using a penile vacuum pump, injections, and using prescribed drugs. HCP expressed that due to lack of understanding among patients in how to take the prescribed medication properly, and emphasized the importance of awareness about informing and following up with patients who are treated for erectile dysfunction. It is important in an earlier stage to talk about sexual dysfunction and alternatives to handle it. HCP experienced that avoiding asking about patients’ sexual health was related to insecurity and lack of knowledge among staff. On the other hand, patients described sexual health as embarrassing and challenging to talk about and, therefore, consciously postponed questions.

You do not talk about this (PC), I mean, there are probably several who cannot talk to anyone about it at all, it is not like talking about a cold, especially if you talk to women. (P14)

When managing disease-related concerns and emotional distress, some men contacted their healthcare providers, used prescribed antidepressant drugs, and talked to family and friends. Patients explained that when they talked openly about their treatment or diagnosis, other men became more open to a conversation. Hot flashes were managed by taking showers, changing clothes, and wearing light clothing, but also by just accepting them and learning to live with them. Patients with decreased strength and tiredness described taking more naps, pauses, walks, and exercising regularly to regain strength and improve fitness. Patients with lymphoedema wore compression stockings with varying adherence, especially during the summer.

#### Formal Support and Unmet Needs

In general, patients described themselves as satisfied with the information and support the healthcare professionals provided. Nevertheless, the patients described being alone with their concerns and side effects and expressed wanting additional support to fulfill their unmet needs. They wished for more continuity and preferred to meet the same nurse or doctor during the follow-up period. Unmet supportive needs were described as needing to remind professionals about follow-ups, not getting feedback on their PSA results, and when calling the healthcare provider, they did not return patients’ calls. Obstacles for patients regarding making contact were exemplified as not wanting to disturb the already-overloaded healthcare system, a fear of being perceived as a problematic or anxious patient, and not knowing whom to call, when, and what would allow a contact.

If I had pushed the question, I would of course have found one of these doctors, but to be honest it is not so easy, you get stuck, and you wonder who to call, I think I have 10 different names so I do not have anyone that I can call. (P16)

HCP also expressed concerns that some patients do not seek help and, therefore, probably do not receive proper support, especially regarding emotional distress and late side effects compared to women with breast cancer. They believed this was due to stereotypical notions of male ideals and staff’s lack of knowledge about prostate cancer.

HCP expressed that information, in general, was a challenging topic. Patients described searching for information online, in books, reading brochures, or calling healthcare providers. The HCP experienced that even though the patients had received information, they might forget. This was exemplified by the patients who described uncertainty about why bodily changes had occurred and uncertainty whether the lack of energy, loss of sexual interest, and tiredness was due to normal aging or was a side effect of treatment. Some patients, who described themselves as well-informed, could still be surprised by side effects and unaware of where to find answers. The HCP believed patients received few consultations to ascertain whether they understood the information they had been given and to validate patients’ concerns and different needs for support. The HCP underlined that it is essential to provide structured follow-up appointments, as it was necessary to repeat tailored oral and written information on the etiology of side effects and how to manage them. HCP underlined that various professions are important during different follow-up phases regarding patients’ concerns and needs. Patients wished for tailored information. Some patients preferred information delivered on an individual basis, as it could be challenging and uncomfortable to ask sensitive questions in a group. At the same time, some expressed it as reassuring to hear other patients talk about their side effects.

…it is about a sense of coherence…sometimes when you meet the patients, you explain why it looks like this, because no one has told them, or they may have told them, but they have forgotten. Just having a sense of coherence makes the situation easier to handle because we teach them to or are trying to teach them to deal with lifelong side effects. (Nurse 1, HCP)

#### Informal Source of Support

Having engaged relatives when being sick was considered important. Support was described as getting help with daily activities like cleaning the house, buying groceries, or helping manage hygiene in the first months after treatment. This kind of support was appreciated and was primarily provided by patients’ wives or daughters. The patients also described how their wives initiated searches for information and contacted healthcare services. Additional support was described as friends or family members calling more often to ask how they were, but this kind of support eventually diminished after a while. Talking to partners, friends, and relatives who had had cancer previously was considered an important source of information and support, especially talking to those who had been treated for prostate cancer was valuable. This made some patients seek no further information or help accepting their situation.

Men with partners or relatives working in healthcare experienced them as good support and information sources to whom they could turn with questions and concerns.

## Discussion

The novelty of the present study is the comprehensive focus on experiences of symptom distress and self-management strategies during the first year after curatively intended treatment for PC from the perspective of both patients and healthcare professionals. The results reveal several areas that could be improved in the supportive care of prostate cancer patients in the aftermath of treatment.

The first year entailed a period in life where patients had to adjust to different bodily functions and appearance changes. The most prevalent symptoms during the first year were changes to urinary, bowel, and sexual function, which has also been previously described.^16–20^ The patient’s experience of lymphoedema was not mentioned in the interviews with HCP and may be explained by that lymphoedema is a condition that is probably underestimated in this group of patients.^32^ Furthermore, unlike in the interviews with HCP, patients described that the bodily changes and the new appearance were sources of distress and affected their perceptions of masculinity, a phenomenon which has also been described previously.^33^

Both patients and HCP saw masculine norms in society as an obstacle when it came to talking about symptoms, and the diagnosis itself was embarrassing and stigmatizing. The HCPs also underlined that existing norms and masculinity being a sensitive topic probably prevent men from receiving proper support. It has been reported that younger men with prostate cancer experience the disease as an “old man” disease related to decreased sexual functioning and fertility.^25^ Masculinity seems to be an important area to recognize in supportive care of patients with prostate cancer, albeit the interviewed HCP in our study underlined that they had concerns that staff in healthcare may not have appropriate education concerning supportive care for patients with PC. It is important to make sure that patients feel safe and comfortable when discussing health issues related to masculinity.^34^

HCP and patients voiced how patients’ symptoms and concerns influenced daily life negatively, primarily in the form of withdrawal from social activities. After treatment ends, patients are at home and left to manage distressing symptoms and side effects^14^ that may negatively affect their quality of life.^19^ In our study, the first three months after the end of treatment appeared to be a vulnerable period, which could be related to less frequent contact with healthcare after hospital-based treatment had ended. During these three months, patients wait for the first post-treatment PSA test result and, at the same time, struggle with the emotional aspects of receiving a prostate cancer diagnosis. Patients also have thoughts about whether the symptom distress would last their entire lives and whether they had made the right decision regarding the type of treatment.^35^

Having engaged family members played an important role in maintaining daily life and contact with health care. In a meta-synthesis that included couples, it was described that partners to patients with prostate cancer are an important supportive source.^36^ Furthermore, the patients in the present study described preferring to talk to men in the same situation, and some of the advice they were given was contradictory to current recommendations and information provided by healthcare professionals. Hence, patients may obtain outdated or incorrect information and advice, which can lead to poor adherence and insufficient self-management and thereby affect symptom distress. Inaccuracies in information about mental health in media, such as YouTube, have recently been noted in a study.^37^ This is noteworthy as patients describe the internet as an information source.

Although information is routinely provided by the health care services to and appreciated by patients, and patients have access to a specific contact nurse, the patients described a need for more information and support. This may be explained by patients forgetting the information they were given, as has been described by patients and HCPs, as well as in the literature.^25^ It has been described earlier that symptoms improve over time, which may hinder patients from seeking information and support due to normalization.^38^ In our study, patients described hesitating to contact healthcare. It is recommended that healthcare providers identify information sources for the patients that are clear, reliable, updated, and tailored to the patient’s preferences.^39^ However, when patients hesitate to contact healthcare, this might keep them from receiving tailored support and information. It is important to recognise the complexity when developing and establishing supportive care, as individual factors (eg, sociodemographic and medical) influence how patients adjust to living with prostate cancer. Patients with higher income, older age, fewer comorbidities, fewer depressive symptoms, and higher health literacy seem to have a more favourable ability to self-manage.^40^ Younger men in this context have been shown to experience more anxiety and depression,^17^,^41^ and poorer life satisfaction.^18^ Men with androgen deprivation and high-risk prostate cancer also seem to have a higher risk for depression^23^,^42^ and even suicide.^42^ Therefore, it is important to consider mental health status in the clinical setting.^42^,^43^ Social support, particularly from friends, can mediate depressive symptoms and body image distress.^44^ Another important issue is that adherence to hormonal treatment is remarkably low (up to 50% of the patients report non-adherence), indicating a lack of sufficient patient education.^45^

The patients in our study clearly described having unmet informational, physical, and emotional needs, as described earlier.^8^,^46^ Additionally, it is already known and was highlighted by the HCPs that patients’ needs for information change over time, and that it is important to provide timely information.^38^,^47^,^48^

It is, therefore, urgently important in an early stage to identify and prevent symptom distress and increase adherence to hormone therapy, as are early actions and implementation of individual supportive care strategies. There is increasing evidence of the utility of nurse-led interventions in cancer care, and oncology nurses and nurse practitioners are well-suited to support survivorship person-centred care.^49^ However, diagnosis and timing are individualized and complicated, and evaluating the effects of nursing interventions in patients with cancer is complex, as they often contain several components.^50^ Patients should be invited to engage, be seen, and become actively incorporated in their care and planning. This is in line with the person-centred approach^51^ and with survivorship care that intends to encourage independence in managing symptoms and side effects.^52^ However, it is important to observe that patients may have positive outlooks after treatment, such as being optimistic and active in finding strategies and appreciating support from family and friends, which is in line with some other studies.^25^,^26^ Positive experiences have been shown to be related to experiences of receiving compassionate care and accessibility to healthcare services.^53^

### Clinical Implications

The present study highlights several areas that should be considered when supporting patients with prostate cancer during the first year after curatively intended treatment. It has been emphasized that there is prime time to develop models of cancer survivorship care integrated with the primary care landscape.^54^ The present study indicates that patients with prostate cancer during the first year after curatively intended treatment are left to navigate for adequate support. During this period of transition, there is a wish to talk about sensitive subjects, and there is a risk of sub-optimal adjustment to the disease and its side effects. A need for increased awareness about supportive care beyond specialist care was also highlighted among healthcare staff, such as more structured follow-up appointments that could enhance individualized care. The variation in minor and severe side effects among men with prostate cancer also calls for developing and adapting models of care that are individually tailored to patients’ needs^55^,^56^ and suitable for primary care.^54^ It has been suggested to implement models for early identification of symptoms using regular digital assessments of customized and meaningful patient-reported outcomes with referral to self-care advice.^57^ A such model based on the present study could include four core components: regular remote assessment of patient-reported outcomes, continuous access to tailored information and self-care advice, health dialogues based on assessments, and, when appropriate, include, informal sources of support for family and friends. Future studies should test person-centred and primary care-focused survivorship models that are introduced immediately after the end of curatively intended treatment.

## Strengths and Limitations

The strength of this study is to elaborate experiences from patients as well as HCP which provided a broad understanding regarding patients’ symptoms and self-management strategies during the first year after curatively intended treatment for prostate cancer. It is important to include perspectives from several stakeholders in the development of relevant and sustainable supportive care interventions.^58^ The choice to perform individual interviews with patients was based on that the topics could be considered sensitive and to achieve their experiences on a personal level.^59^ The choice of performing a focus group interview with the HCP was based on gaining data on interaction and discussion between the participants.^60^ One limitation with focus group interviews may be that not all get the opportunity to speak, but all participants were active and interacted during the interview.

A limitation is the lack of a physician as a participant in the focus group interview; those invited declined participation due to a heavy workload at the scheduled time of the interview. However, all the HCPs involved had a long experience working with support to patients with prostate cancer. Another limitation is that only one pilot study was performed. The patient had no problems understanding the questions in that interview, and there were no problems during the following interviews. The open question gave the patients the opportunity to formulate their answers freely, and with support from the follow-up questions, data richness was achieved. Six patients were interviewed by telephone due to the COVID-19 pandemic. In Sweden, there was no lockdown; the recommendation was social distancing. There were no differences in the richness and content of the data between the face-to-face and telephone interviews. There were no data in the telephone interviews that were explicitly related to the COVID-19 pandemic. Hence, the pandemic seemed not to have influenced the patients regarding their prostate cancer diagnosis at the time of interviews.

The patients in our interviews varied in age and treatment, but most were in a relationship. Thus, more variations in civil status among the participants might have yielded additional data regarding supportive needs. Patients were recruited from one university hospital in Stockholm, Sweden, which is considered a limitation. How patients may experience their care can be related to routines at the hospital and the healthcare staff. However, the study took place at one of Sweden’s largest hospitals, including both rural and urban areas, and two clinics with different staff: one oncology and one urology clinic. After the performed focus group interview and 17 individual interviews, the research group discussed further data collection and decided that data saturation had been reached. The analysis process was characterized by flexibility and was continuously discussed within the research group to reach credibility and improve confirmability.^61^

## Conclusions

Patients with prostate cancer were shown to have lingering symptom distress and challenging physical and psychological transitions during the first year after the end of treatment, and their self-management strategies varied. Patients prefer continuous, individualized, and updated support in managing their individual side effects and concerns. Our results indicate that support should be offered timely and tailored immediately after curatively intended treatment.

## Data Sharing Statement

The data supporting this study’s findings are available from the corresponding author on reasonable request.
